# Automated classification of choroidal neovascularization, diabetic macular edema, and drusen from retinal OCT images using vision transformers: a comparative study

**DOI:** 10.1007/s10103-024-04089-w

**Published:** 2024-05-27

**Authors:** Said Akça, Zeynep Garip, Ekin Ekinci, Furkan Atban

**Affiliations:** Department of Computer Engineering, Sakarya University of Applied Sciences, Sakarya, Turkey

**Keywords:** Transformer, ViT, Classification, Retinal Diseases, Diagnosis, OCT

## Abstract

Classifying retinal diseases is a complex problem because the early problematic areas of retinal disorders are quite small and conservative. In recent years, Transformer architectures have been successfully applied to solve various retinal related health problems. Age-related macular degeneration (AMD) and diabetic macular edema (DME), two prevalent retinal diseases, can cause partial or total blindness. Diseases therefore require an early and accurate detection. In this study, we proposed Vision Transformer (ViT), Tokens-To-Token Vision Transformer (T2T-ViT) and Mobile Vision Transformer (Mobile-ViT) algorithms to detect choroidal neovascularization (CNV), drusen, and diabetic macular edema (DME), and normal using optical coherence tomography (OCT) images. The predictive accuracies of ViT, T2T-ViT and Mobile-ViT achieved on the dataset for the classification of OCT images are 95.14%, 96.07% and 99.17% respectively. Experimental results obtained from ViT approaches showed that Mobile-ViT have superior performance with regard to classification accuracy in comparison with the others. Overall, it has been observed that ViT architectures have the capacity to classify with high accuracy in the diagnosis of retinal diseases.

## Introduction

Age-related macular degeneration (AMD) occurs in people aged 50 years and older and is one of the main reasons of central vision loss and permanent blindness worldwide [1]. AMD affects the central region of the retina known as the macula and is classified as dry or wet AMD. Drusen is a typical example of dry AMD. Containing various proteins associated with inflammation and lipids, Drusen are extracellular materials located between the retinal pigment epithelium (RPE) and Bruch's membrane, causing focus elevations in the RPE [2]. Choroidal neovascularization (CNV) is wet-type AMD. CNV is defined as the growth of new blood vessels emerging from the choroid into the sub-RPE space through a break in Bruch's membrane [3]. Diabetic macular edema (DME) occurs as a result of deterioration of the neurovascular structure in people with diabetes. DME is one of the primary reasons of vision loss in people aged 20–79 worldwide [4]. Nowadays, early diagnosis of these diseases in terms of ophthalmology and rapid response to treatment are very important [5].

OCT is a biomedical imaging technique that uses the coherent properties of light for the early diagnosis and treatment of AMD and DME-type retinal diseases. OCT is also critical as it is the preliminary in diagnostic imaging [6]. OCT provides a cross-sectional view of the retina and lesions to diagnose and follow AMD and DME with high resolution and non-invasive imaging [7].

It is very difficult to make the classification accurately with the computer perspective. However, the increase in the number of patients in medical centers and the fact that there are different opinions among specialists during the diagnosis of diseases also make it difficult to classification. However, an important advantage of classification models is; It can reach the right result in the fastest and most accurate way by eliminating the speed, time and accuracy problems caused by the increasing number of patients and different opinions during the detection. Therefore, deep learning-based classification models can be used, in which OCT images are automatically classification.

Most of the classification studies in the literature include classical deep learning [8–10] and traditional machine learning algorithms [11]. Fu et al. proposed a deep learning based automatic detection model for the presence of angle closure on (Anterior segment optical coherence tomography) AS-OCT images obtained from a time-domain OCT. As a result, it was reported that the proposed method reached 90% ± 0.02 sensitivity [12]. In another study conducted for the detection of macular diseases and segmentation of lesion areas, Liu et al. used one-stage attention-based Convolutional Neural Network (CNN). When the results were examined for four different classes, it was seen that an accuracy value of 93.6% for the CNV class, 94.8% for the DME class, 94.6% for the Drusen class, and 97.1% for the Normal class were achieved [13]. Sunija et al. proposed a CNN- Long Short-Term Memory (CNN-LSTM) for glaucoma detection from raw Spectral-Domain-OCT (SD-OCT) images and an accuracy value of 99.64% was obtained [14]. Altan et al. reported an accuracy value of 99.20% with the DeepOCT model for classification of macular edema [15]. Lo et al. presented a federated learning framework to enable the classification of diabetic retinopathy by OCT and OCT angiography (OCTA) images. Based on the study's findings, AUROC values of 95.4% and 96% were acquired for OCT and OCTA respectively [16]. Naz et al. classify OCT images to differentiate individuals with DME from normal ones using the Support Vector Machine (SVM) classifier and an accuracy value of 79.65% was reported [17]. Venkatraman et al. presented a machine learning-based method to detect abnormalities in the retinal layer with the SVM classifier and an accuracy value of 87.98 was obtained [18]. Sugmk et al. classified abnormalities in retinal diseases as AMD and DME for OCT images using an image segmentation technique. [19]. Das et al. devised deep multiscale fusion CNN (DFM-CNN). As a result of the study, 96.03% and 99.60% accuracy values were obtained for the University of California San Diego (UCSD) and the Noor Eye Hospital (NEH) datasets, respectively [20]. Najeeb et al. classified retinal abnormalities in retinal OCT images using a single layer CNN structure to with an accuracy value of 95.62% [21]. Serener et al. evaluated the performance of the ResNet and AlexNet architectures to accurately detect dry age-related macular degeneration and wet age-related macular degeneration [22]. Kang et al. classified pachychoroid and non-pachychoroid eyes based on OCT-B scan images using ResNet50 and InceptionV3 models and achieved 96.1% and 95.25% accuracy values, respectively [23]. Perdomo et al. presented an automatic image analysis method called a custom OCT-NET model based on CNNs for the detection of DME. As a result of the study, it was shown that the CNN based OCT-NET model gave a successful result with an accuracy of 93.75% [24]. Kim et al. used VGG-16 architecture for automatic segmentation and classification of retinal layers [25].

The novelty of in this study can be summarized as a comparative analysis and investigation of the effect of different transformer methods on success. Verification of ViT's diagnostic efficacy for various retinal disorders such as CNV, drusen and DME is infrequent.

The main contributions of this study are as follows:

- We point to an automatic diagnosis system to avoid delays in the diagnosis process brought about by the difficulty of meaningful analysis of images by medical professionals as a result of the increasing number of daily OCT scans taken in medical centers.
- By going beyond the existing methods in the literature, we show the success of the use of transformers in the classification of retinal diseases.
- We reveal the idea of the applicability of the method used for the analysis and classification of different biomedical images.
- We demonstrate the success of transformers in terms of calculation efficiency and accuracy.
- To show the success of the evaluation results of the proposed model, it is compared with the results obtained in the literature.
- Comparative studies have been conducted with ViT, T2T-ViT and Mobile-ViT and on retinal OCT images. There are no similar comparative analyses, according to our best knowledge.

## Materials and methods

### Dataset

We used publicly available open-access Labeled Optical Coherence Tomography (OCT) and Chest X-Ray Images dataset for experimenbts [26]. Original dataset has 84,495 OCT images divided into four categories: CNV, drusen, DME and normal. The distribution among the classes in the dataset is unequal. The success of the predictive model requires that the amount of data used to solve the retinal diseases classification problem be established with equal or approximate data sizes for each class label. A total of 34,464 data are taken, with 8,616 for each class used in the training and testing process. The total number of images in the original dataset and the number of images used are given in Table 1 and characteristics of patients are given Table 2.

**Table 1:** Total and number of images used in the dataset

Label	Total Images	Experimental Images (Training)	Experimental Images (Testing)
CNV	37,455	8,616	242
Drusen	8916	8,616	242
DME	11,598	8,616	242
Normal	26,615	8,616	242

**Table 2:** Characteristic of patients whose OCT images were in the original dataset

Characteristic	CNV	Drusen	DME	Normal
Number of Patients	791	713	709	3548
Mean Age (Years)	83 (Range: 58–97)	82 (Range: 40–95)	57 (Range: 20–90)	60 (Range: 21–86)
Gender				
Male	54.2%	44.4%	38.3%	59.2%
Female	45.8%	55.6%	61.7%	40.8%
Ethnicity				
Caucasian	83.8%	85.2%	42.6%	59.2%
Asian	6.3%	8.6%	23.4%	21.1%
Hispanic	8.3%	4.9%	23.4%	10.2%
African American	2.1%	1.2%	4.3%	1.4%
Mixed or Other	0%	0%	10.6%	7.5%

**Fig. 1:**
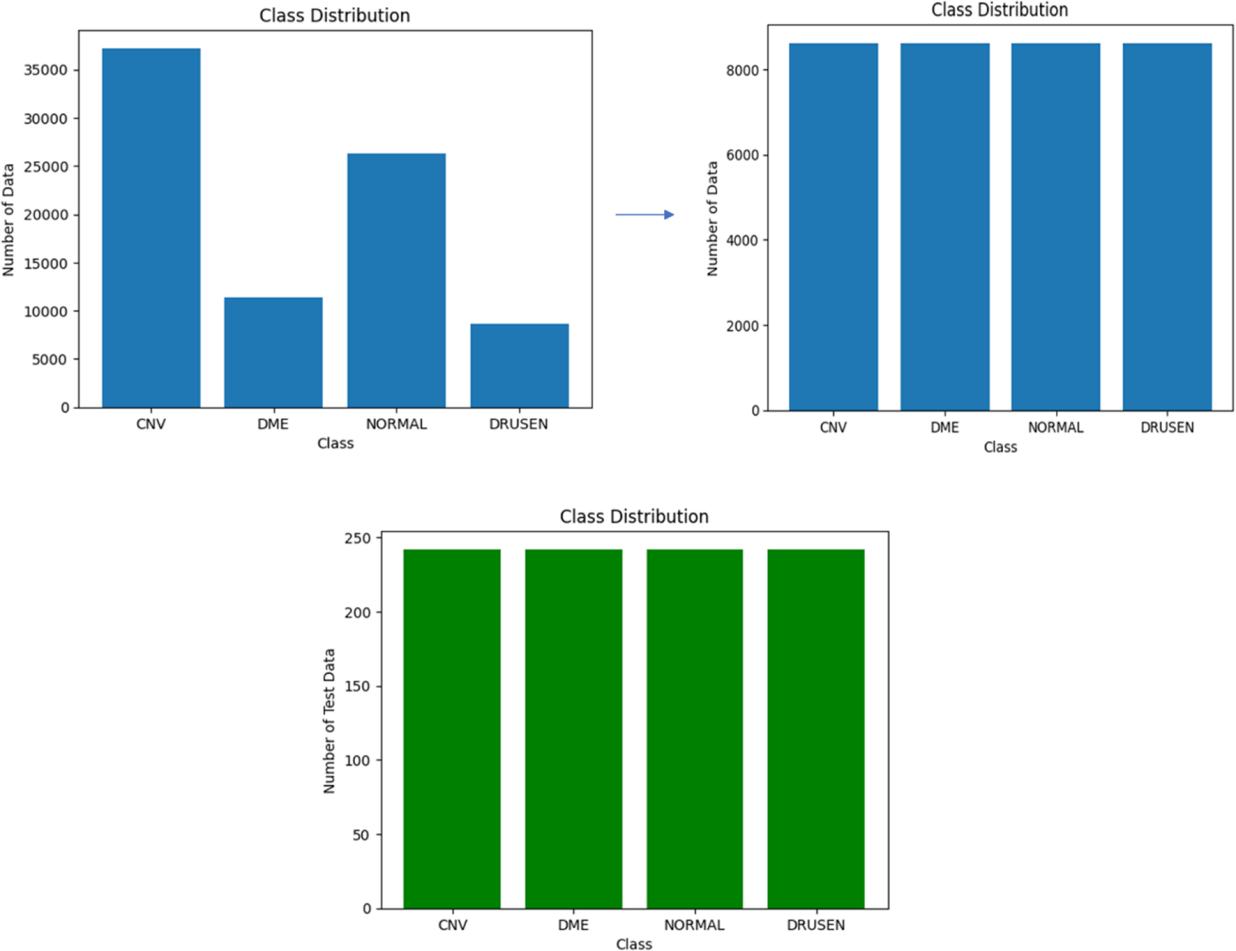
Class distribution used in the proposed models

The main reasons of problems by deep learning models is that they may encounter problems with overfitting or underfitting during training. The validation data obtained by dividing the training data is not used for training, it is used to measure the performance of the model during training. For this reason, within the scope of the study, it is aimed to create validation data with a split ratio of 0.1 and to use the results obtained with these data in the back propagation process to ensure that they directly affect the learning process. It was determined as a total of 968 test data with 242 data from each class. The distribution of training and test data used in the study is shown in Fig. 1.

### Proposed model

**Fig. 2:**
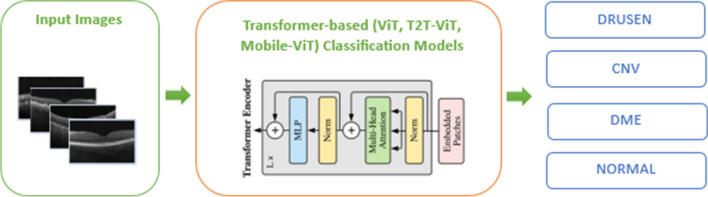
The framework of the proposed transformer based approaches for retinal disease detection

To evaluate the classification effects of different networks for optical coherence tomography images we use three kinds of transformer based network architecture based on the attention mechanism: ViT, T2T-ViT, Mobile-ViT. The transformer models have been supported with sufficient data and more accurate predictions have been pointed out. Figure 2 is shown the framework of the proposed approaches for retinal disease detection.

### Vision transformer model

**Fig. 3:**
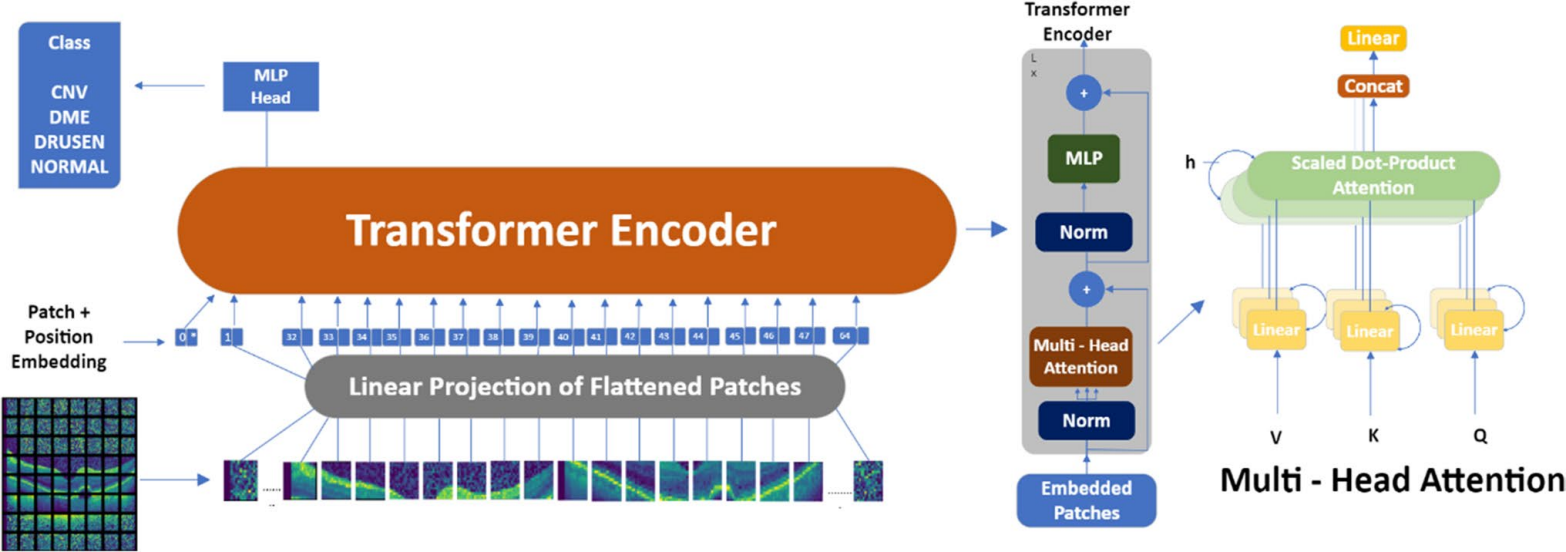
ViT architecture

Vision Transformer (ViT) model essentially creates the image it takes as an input as a series of image patches by processing it. Accordingly, it predicts the class label. The concept of pixel is the most basic unit for the analysis of each image. However, considering the computational relations for each pixel, large costs may arise in terms of computational efficiency. Instead, ViT significantly reduces the semantic relationships between pixels by breaking down the image into patches, thus aiming to reduce costs. Patch are placed in a row. These embedded embeds are learnable vectors. The ViT structure obtains it by multiplying each segment by the embedding matrix, ensuring that it takes a linear order. The extracted results are transferred to the transformer encoder by position embedding. Transformer encoder structure includes a multi-head self-attention layer, multi-layer perceptron layer and norm layer. Here, the self-attention layer ensures that information is embedded throughout the image. The model learns the training data to be able to reconstruct the structure of the image. The MLP layer applies the classification header for image classification [27]. The ViT architecture model is shown in Fig. 3.

**Fig. 4:**
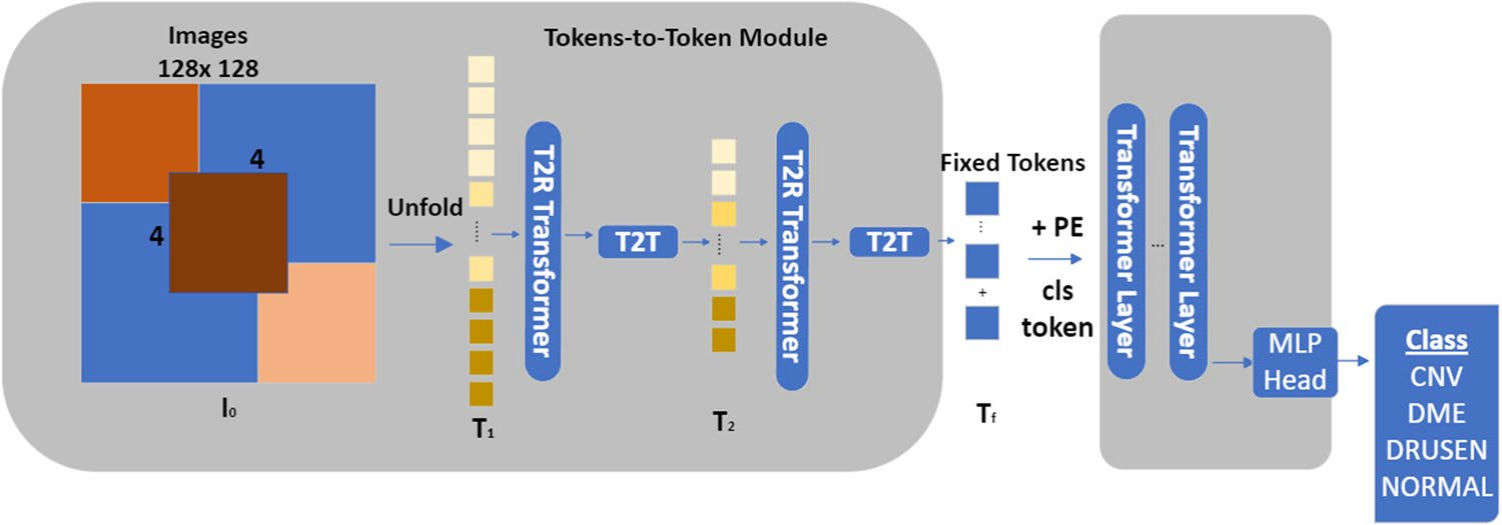
T2T-ViT architecture

The T2T-ViT model is based on the ViT structure. The T2T-ViT model works by adopting the image as a language and encoding the image pixels as tokens. Tokens are the biggest feature that distinguishes T2T-ViT from ViT [28]. T2T-ViT architecture model is shown in Fig. 4.

**Fig. 5:**
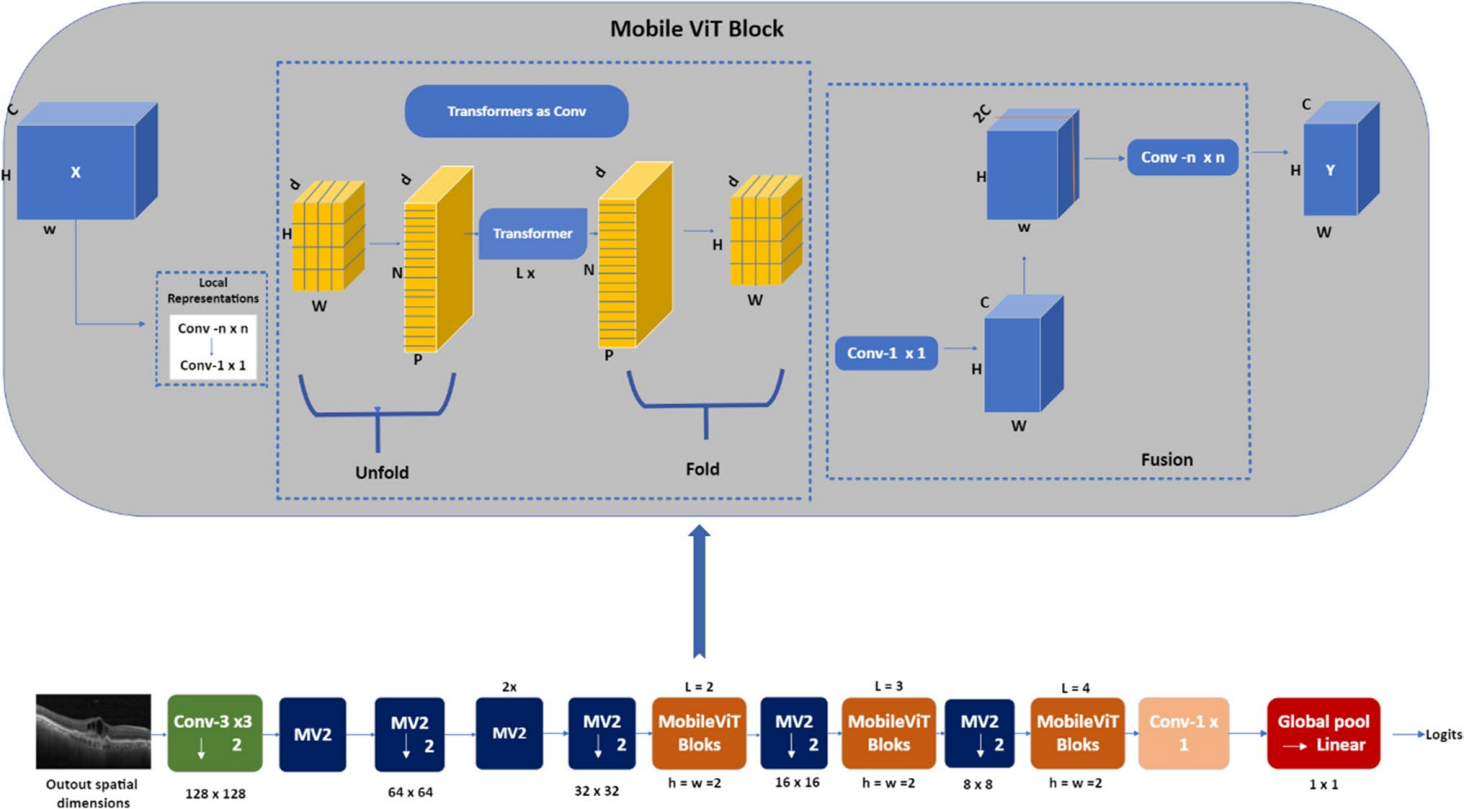
Mobile-ViT architecture

Mobile-ViT represents a lightweight ViT structure. It covers spherical representations learning with transformers as convolutions. Learning global representations using transformers as convolution is the main goal. As a result, we are able to implicitly add convolution-like features into the network, learn representations using straightforward training recipes, and easily integrate Mobile-ViT with downstream architectures [29]. Mobile-ViT architecture model is shown in Fig. 5.

## Experimental results

In the proposed study, the classification performance of transformer-based classification methods on a dataset with four different class namely CNV, DME, Drusen and Normal retina has been evaluated. To measure the performance of the model, precision, recall, accuracy and F1-score are used. To explain through a class, where true positive (TP) is the number of truly classified Drusen images. False positive (FP) shows the number of images predicted as Drusen but not Drusen. False negative (FN) is the incorrect classification of images as CNV, DME or Normal retina. True negative (TN) is the number of correctly classified not Drusen images. The formulas of performance metrics in terms of TP, FP, FN and TN and entropy are given with Eq. (1–5).

1 $$Accuracy=\frac{TP+TN}{TP+FP+FN+TN}$$

2 $$Precision=\frac{TP}{TP+FP}$$

3 $$Recall=\frac{TP}{TP+FN}$$

4 $$F1Score=2X\frac{PrecisionXRecall}{Precision+Recall}$$

5 $$Categoricalcrossentropy=-\sum\limits_{i=1}^iq\left(y_i\right)log\left(p\left(y_i\right)\right)$$

In this direction, we apply three different approaches. First, the classification process was carried out with the ViT model. To apply the model, a hyper parameter selection analysis was performed and parameter values were determined. Keeping the learning rate value too high can mean too much influence from the data. Choosing a small value will cause the training to take too long. This will negatively affect the training process in terms of speed and cost. For this reason, the learning rate value was chosen as 0.001 among the three different methods used in the study for optimum results. Adaptive algorithms run a faster process than SGD. Therefore, for all methods, AdamW, which has a dynamic structure, was chosen as the optimizer. Additionally, Categorical Cross Entropy was selected as loss function and 100 was used as the epoch value. The fact that the epoch value was very high showed that the learning status of the model decreased after a certain point.

**Fig. 6:**
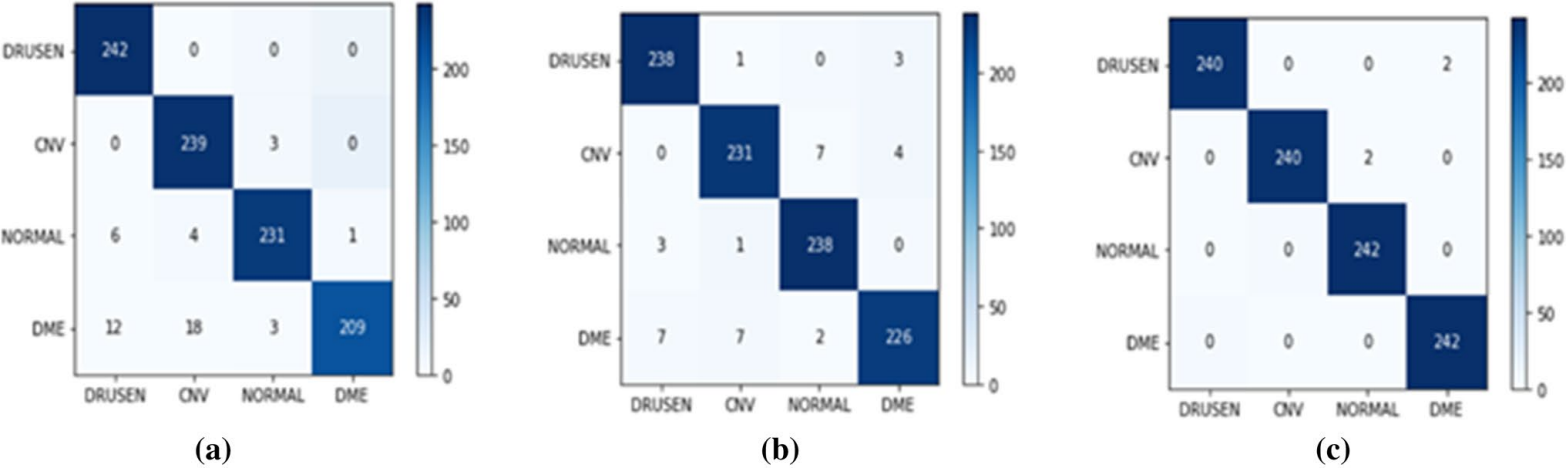
Confusion matrices for all transformer based classifiers (**a**) ViT (**b**) T2T-ViT (**c**) Mobile-ViT

When the results obtained from 968 test samples of 128x128x3 dimensions were examined, an accuracy value of 95.14% was obtained with ViT. As a result of the T2T-ViT approach which eas applied in the second stage, an accuracy value of 96.07% was obtained. In the last phase of the study, we apply the Mobile-ViT approach for retinal disease classification. Accordingly, an accuracy value of 99.17% was obtained. The performance metrics obtained for each class label with all the approaches applied are shown in Table 3. The confusion matrices obtained by the three different approaches proposed are shown in Fig. 6.

**Table 3:** Result of performance metrics for all transformer-based classifiers

Algorithms	Class	Precision (%)	Recall (%)	${F}_{1}$ Score (%)	Accuracy (%)
ViT	DRUSEN	93.07	100	96.41	95.14
	CNV	91.57	98.76	95.02	
	NORMAL	97.46	95.45	96.44	
	DME	99.52	86.36	92.47	
T2T—ViT	DRUSEN	95.96	98.34	97.13	96.07
	CNV	96.25	95.45	95.84	
	NORMAL	96.35	98.34	97.33	
	DME	96.99	93.38	95.15	
Mobile ViT	DRUSEN	100	99.18	99.58	99.17
	CNV	100	99.18	99.58	
	NORMAL	99.18	100	99.58	
	DME	99.18	100	99.58

In terms of classifying OCT images into four groups, the suggested approaches performed the best when compared to other systems as shown in Table 4. Mobile-ViT model achieves a high classification rate when compared with alternative methods. Transformer based models classifies the four retinal diseases more accurately using the OCT image dataset,

**Table 4:** Performance comparison in literature

Reference	Year	Models	Accuracy (%)
In this study	2024	ViT	95.14
		T2T—ViT	96.07
		Mobile ViT	99.17
Rajagopalan et al. [30]	2021	CNN	95.70
Paul et al. [31]	2020	Deep Ensemble Network	98.53
Kim and Tran [32]	2020	ResNet152	98.90
Kermany et al. [33]	2018	Inception V3	96.60

## Conclusions

In the medical sector, the need for automatic diagnosis systems is increasing day by day. With the increasing human population, the increasing patient density makes it difficult for experts to make quick and accurate decisions. As a result of such reasons, it has been tried to find a solution to this problem with different artificial learning approaches.

In this study, we focus on the classification of retinal diseases with the transformer approach. The novelty of this study can be summarized as a comparative study examining the impact of transformers-based architectures. Comparative studies are conducted with ViT, T2T-ViT and Mobile-ViT on the retinal OCT images dataset. To the best of our knowledge, similar comparative analyzes are not available. The results indicate the idea of concrete applicability of the study in the health sector. The accuracy value of 99.17% and other results obtained with the Mobile-ViT structure shows that the Transformer structure based on the attention mechanism gives successful results for object classification, which is the subject of computer vision. Thus, we reveal the applicability of transformer approximations to the classification process by capturing spherical and wider property relationships. The results show that the transformer structure can be used especially in terms of medical image classification, apart from different deep learning approaches in the literature. At the same time, it has been shown that the structure of the transformer, contrary to what is thought, is not only successful for training based on large data sizes but also for smaller size datasets. Transformer-based classifiers have emphasized that they provide high speed and classification performance with a lightweight architecture for small-scale datasets by reducing the number of parameters.

Standards and frameworks for image quality assessment for OCT images are important for accurate imaging-based diagnosis. In future studies, we plan to apply these approaches to datasets with images at different magnification levels to evaluate image quality. Additionally, new transformer-based classifier designs will be a new research method.
